# Hierarchical Reproductive Allocation and Allometry within a Perennial Bunchgrass after 11 Years of Nutrient Addition

**DOI:** 10.1371/journal.pone.0042833

**Published:** 2012-09-11

**Authors:** Dashuan Tian, Qingmin Pan, Matthew Simmons, Hada Chaolu, Baohong Du, Yongfei Bai, Hong Wang, Xingguo Han

**Affiliations:** 1 State Key Laboratory of Vegetation and Environmental Change, Institute of Botany, Chinese Academy of Sciences, Beijing, China; 2 Graduate University of Chinese Academy of Sciences, Beijing, China; 3 Agriculture and Natural Resources Department, University of Minnesota-Crookston, Crookston, Minnesota, United States of America; 4 Xilingol Vocational College, Inner Mongolia Autonomous Region, Xilinhot, China; 5 Semiarid Prairie Agricultural Research Centre, Agriculture and Agri-Food Canada, Swift Current, Saskatchewan, Canada; University of Alberta, Canada

## Abstract

Bunchgrasses are one of the most important plant functional groups in grassland ecosystems. Reproductive allocation (RA) for a bunchgrass is a hierarchical process; however, how bunchgrasses adjust their RAs along hierarchical levels in response to nutrient addition has never been addressed. Here, utilizing an 11-year nutrient addition experiment, we examined the patterns and variations in RA of *Agropyron cristatum* at the individual, tiller and spike levels. We evaluated the reproductive allometric relationship at each level by type II regression analysis to determine size-dependent and size-independent effects on plant RA variations. Our results indicate that the proportion of reproductive individuals in *A. cristatum* increased significantly after 11 years of nutrient addition. Adjustments in RA in *A. cristatum* were mainly occurred at the individual and tiller levels but not at the spike level. A size-dependent effect was a dominant mechanism underlying the changes in plant RA at both individual and tiller levels. Likewise, the distribution of plant size was markedly changed with large individuals increasing after nutrient addition. Tiller-level RA may be a limiting factor for the adjustment of RA in *A. cristatum*. To the best of our knowledge, this study is the first to examine plant responses in terms of reproductive allocation and allometry to nutrient enrichment within a bunchgrass population from a hierarchical view. Our findings have important implications for understanding the mechanisms underlying bunchgrass responses in RA to future eutrophication due to human activities. In addition, we developed a hierarchical analysis method for disentangling the mechanisms that lead to variation in RA for perennial bunchgrasses.

## Introduction

Reproductive allocation (RA) is a core component of plant life history. Different patterns of RA usually reflect different plant strategies that are shaped by long-term natural selection [1]. Under changing environments, however, plants can modify their allocation patterns to cope with environmental constraints. Therefore, variation in RA plays a pivotal role for plant adaptation to environmental changes. Elucidating the mechanisms responsible for RA variation is of great importance for predicting plant responses to future environmental changes.

Because plant growth is an allometric process, any factor that affects plant size may also influence plant RA. This size-dependent effect on plant RA has been observed frequently [2]–[5]. In contrast, reports also show that plant RA is affected by environmental factors in a size-independent manner [6], [7]. Therefore, separation of size-dependent from size-independent effects is critical for understanding the underlying mechanisms leading to variations in plant RA.

The theory of allometry provides a useful framework for disentangling the size-dependent and size-independent effects on plant RA variation [3], [4], [7]–[9]. Allometric relationships are generally described by the equation y  =  β xα, or more commonly Log (y)  =  Log (β) + α Log (x); where x and y are vegetative (V) and reproductive (R) biomass, respectively, α is the allometric slope, and β is a regression constant. There are three types of allometric slope of R-V curves that represent three mechanisms responsible for variation in RA. First, the R-V relationship exhibits a fixed allometric relationship as indicated by a constant allometric slope, suggesting that the size-dependent effect plays a dominant role in variation of plant RA [9]. Second, the R-V relationship is a plastic allometric one with varied allometric slopes, suggesting that both the size-dependent and size-independent effects are important for plant responses in RA [6], [7]. Third, the R-V relationship does not follow an allometric trajectory, suggesting that the size-independent effect plays a dominant role in variation of plant RA [10]. Examining the R-V relationships can help us understand the role of size-dependent versus size-independent effects in adjusting plant reproductive investment.

Most studies on reproductive allocation and allometry have focused on RA at the whole plant level [11]–[15]. However, reproductive investment is a hierarchical process [16]. For a perennial bunchgrass, some buds develop into reproductive tillers while others develop into vegetative ones. Assimilates are allocated to either vegetative tillers or reproductive tillers. The individual-level reproductive investment refers to the mass of an individual allocated to reproductive tillers. The mass of a reproductive tiller is further allocated to either reproductive organ (spike) or vegetative organs (e.g. stem, leaf and root). The tiller-level reproductive investment refers to the mass of a tiller allocated to its spike. Following this line, the spike can be divided into seeds and non-seed components, and the spike-level reproductive investment refers to the mass of a spike allocated to its seeds. Therefore, the final reproductive investment at the whole plant level for a bunchgrass depends on the allocation of an individual's biomass to its reproductive tillers, the allocation of a tiller's biomass to its spike and the allocation of a spike's biomass to its seeds. A perennial bunchgrass may adjust its reproductive investment at different hierarchical levels. For example, it can increase (or decrease) the number of reproductive tillers at the individual level [17] or change the number of seeds by selective abortion at the spike level [18]. It is of great importance to explore the RA patterns at each hierarchical level to better understand the whole process of plant reproduction, especially under changing environments. Obeso (2004) has suggested that plant RAs and allometric relationships should be analyzed by a bottom-up method along hierarchical levels [19]. However, to the best of our knowledge, there has been no study to synchronously examine plant reproductive allocation and allometry along hierarchical levels within a plant population.

**Figure 1:**
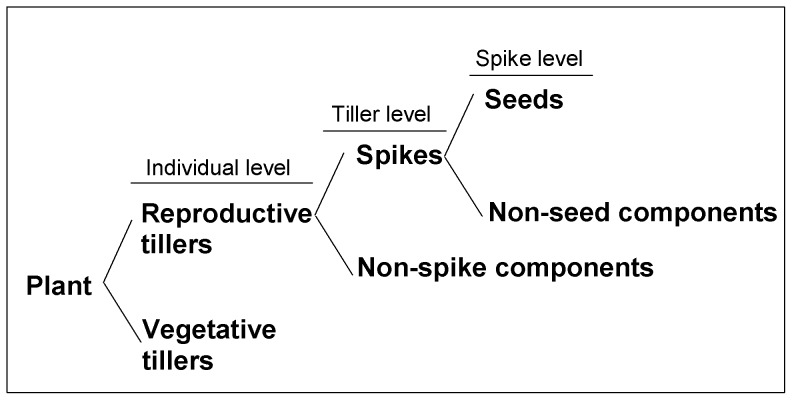
Reproductive modules of *A. cristatum*. At the individual level, an individual was divided into vegetative tillers and reproductive tillers. At the tiller level, a reproductive tiller was divided into a spike and non-spike components. At the spike level, a spike was divided into seeds and non-seed components.

Eutrophication has become a fatal threat to plant diversity, and will profoundly influence the structure and function of grassland ecosystems [20]–[22]. Examining species-level responses in RA is essential for our understanding of the causes for community-level changes [4]. To date, most studies examining responses of plant RA to nutrient addition have been conducted in greenhouse with relatively short duration. Varying results have been reported such that an increase [23], a decrease [24] and no change [25] in plant RA have been observed. However, there has been little information about RA responses at the species-level in natural communities [4]. As the semi-arid grassland in Inner Mongolia is mainly limited by nitrogen and phosphorus [21], [26], it is expected that long-term nutrient addition would have significant effects on species-level responses in RA in this ecosystem. We examined reproductive allocations and analyzed relationships between vegetative and reproductive biomass in *Agropyron cristatum*, a dominant bunchgrass, at individual, tiller and spike levels (Figure 1) in a field experiment with eleven years of nutrient addition. Specifically, we addressed the following questions: 1) How do RAs of *A. cristatum* at different hierarchical levels differ in response to rates of nutrient addition? 2) What is the relative importance of size-dependent versus size-independent effects on the variations in RA at different hierarchical levels?

**Figure 2:**
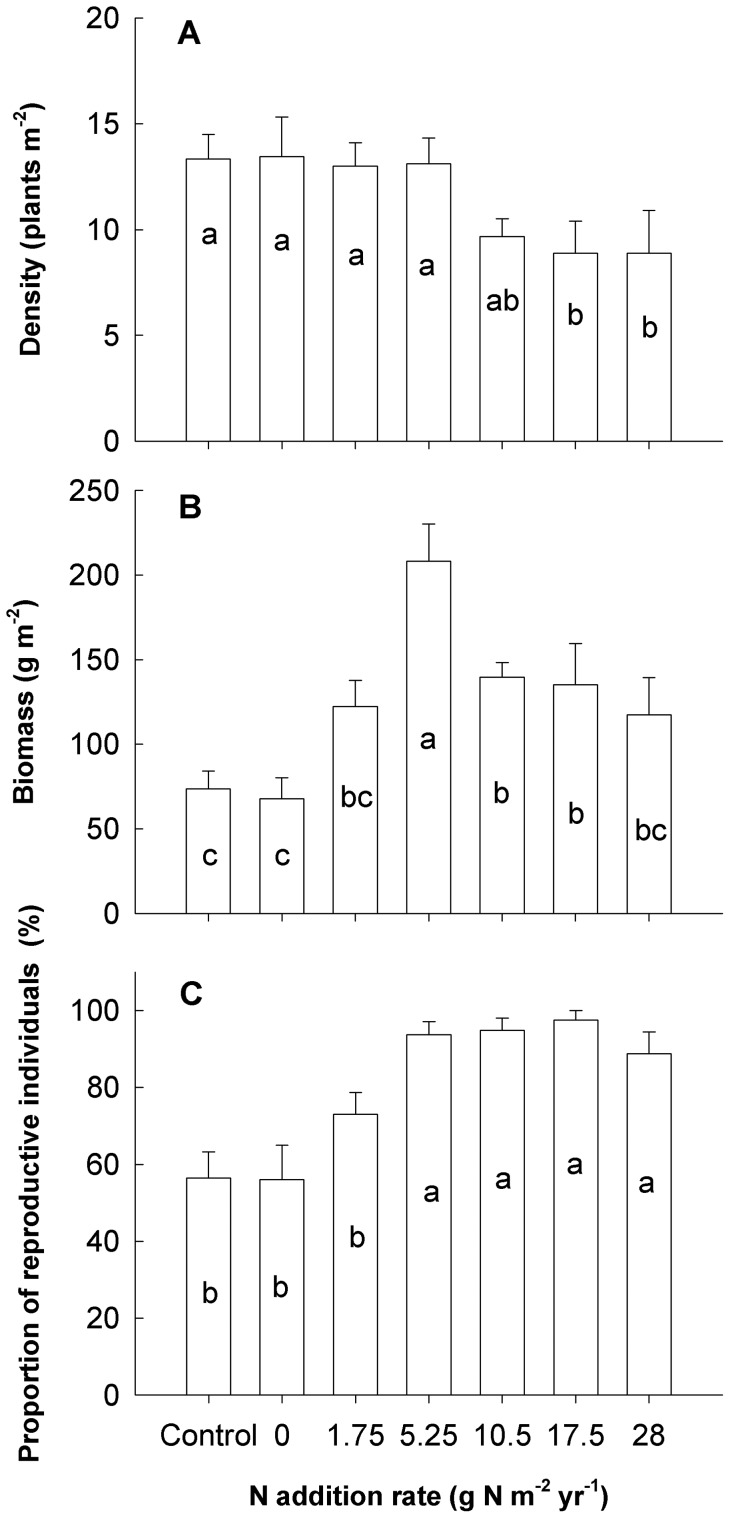
Effects of nutrient addition on density (A), biomass (B) and proportion of reproductive individuals (C) of *A. cristatum* at the population level. The height of the bar for each treatment is the average of nine replicates (error bars indicate s.e.). Bars followed by the same letter are not significantly different according to Duncan's multiple range tests at *P* = 0.05.

**Figure 3:**
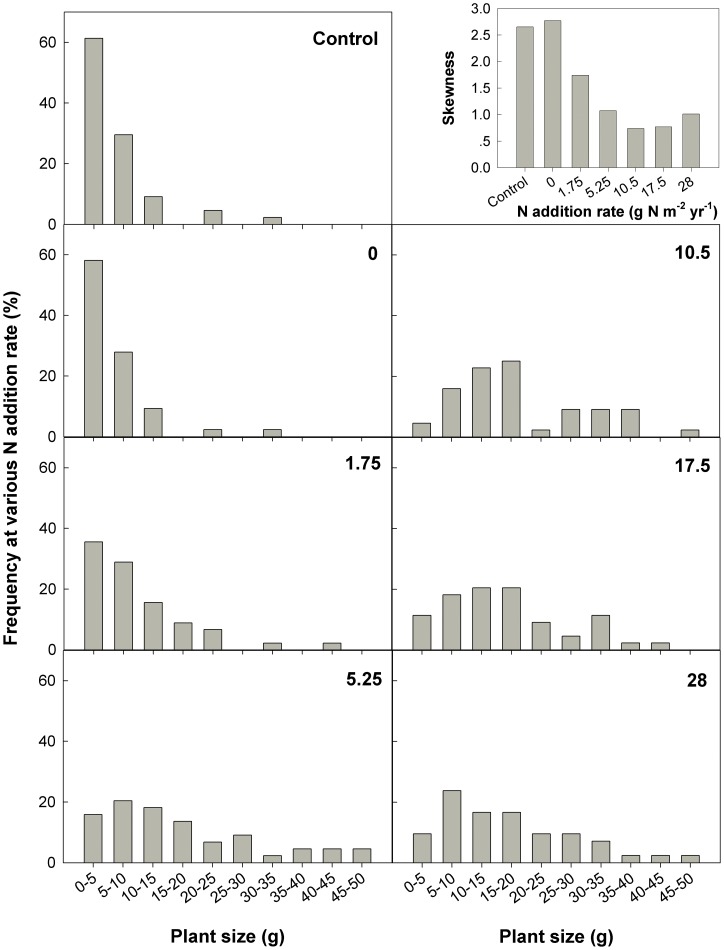
Size distribution of *A. cristatum* individuals in response to nutrient addition rates.

## Materials and Methods

### Site description

This study was conducted at a permanent enclosure in a grassland community in Inner Mongolia Autonomous Region, China (E 116°42′, N 43°38′; elevation 1250 m a.s.l.), which had been fenced by the Inner Mongolia Grassland Ecosystem Research Station (IMGERS) since 1999. This station is a key member of the Chinese Ecological Research Network (CERN) and is managed by the Institute of Botany, Chinese Academy of Sciences. In the experimental area, mean annual precipitation is 346.1 mm, of which 60–80% occurs during the plant growing season from May to August. Mean annual temperature is 0.3°C with a minimum monthly temperature of −21.6°C in January and a maximum monthly temperature of 19.0°C in July. The soil is dark chestnut. Prior to nutrient addition, the plant community was dominated by a C_3_ bunchgrass (*Stipa grandis*) and a C_4_ bunchgrass (*Cleistogenes squarrosa*). After 11 years of nutrient addition, *Agropyron cristatum* has become the dominant species, mainly due to its increase in height.

**Figure 4:**
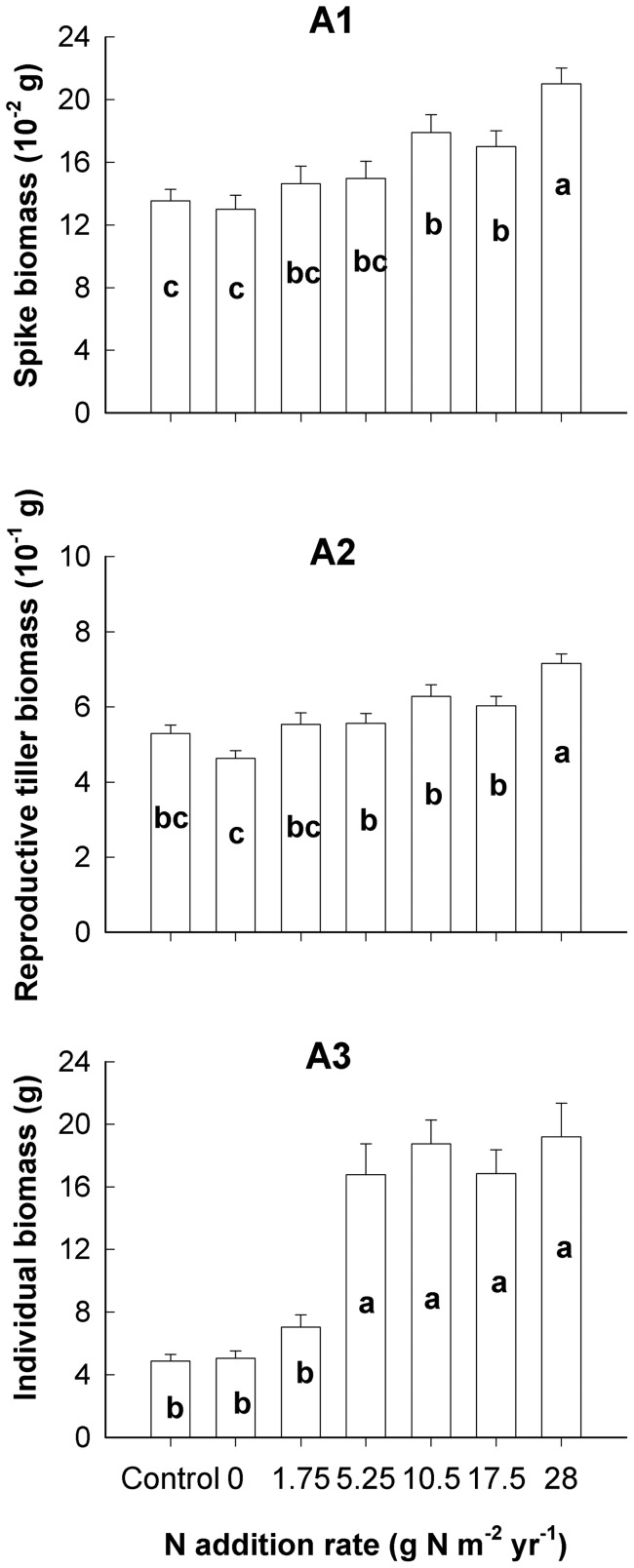
Effects of nutrient addition on biomass of *A. cristatum* at the spike (A1), tiller (A2) and individual (A3) levels. Biomass for each treatment is the average of 45 replicates (error bars indicate s.e.). Bars followed by the same letter are not significantly different according to LSMEANS at *P* = 0.05.

**Table 1:** F values and *P* values for variance analysis by a nested mixed model with N treatments as fixed factors and replicate and plant hierarchical level as random factors.

Response variables	Numerator df	Denominator df	F value	*P* value
Individual biomass (g)	6	55.5	16.63	<.0001
Tiller biomass (10^−1^ g)	6	57.6	6.66	<.0001
Spike biomass (10^−2^ g)	6	56.6	7.86	<.0001
Spike-level RA (%)	6	57.5	1.08	0.3854
Tiller-level RA (%)	6	54.4	2.35	0.0457
Individual-level RA (%)	6	53.4	4.87	0.0005
Final RA at the whole plant level (%)	6	56.6	1.70	0.1376

### Experimental design

In 1999, the 120 m ×70 m study site was fenced off from large animals. The nutrient addition experiment began in 2000 and included 7 treatments, each with 9 replications. Plots (5 m ×5 m) were arranged following a randomized block design. Six levels of nitrogen addition (0, 1.75, 5.25, 10.5, 17.5 and 28.0 g N m^−2^ yr^−1^) were created by adding NH_4_NO_3_ to plots at the beginning of July every year. To ensure that N was the only limiting element, the following elements were also added: P (10 g P_2_O_5_ m^−2^ yr^−1^), S (0.2 mg m^−2^ yr^−1^), Zn (190 µg m^−2^ yr^−1^), Mn (160 µg m^−2^ yr^−1^) and B (31 µg m^−2^ yr^−1^). In addition, there were 9 control plots in which no nutrient was added. In total, there were 63 plots with 54 plots being fertilized every year and 9 plots never receiving any nutrients.

**Figure 5:**
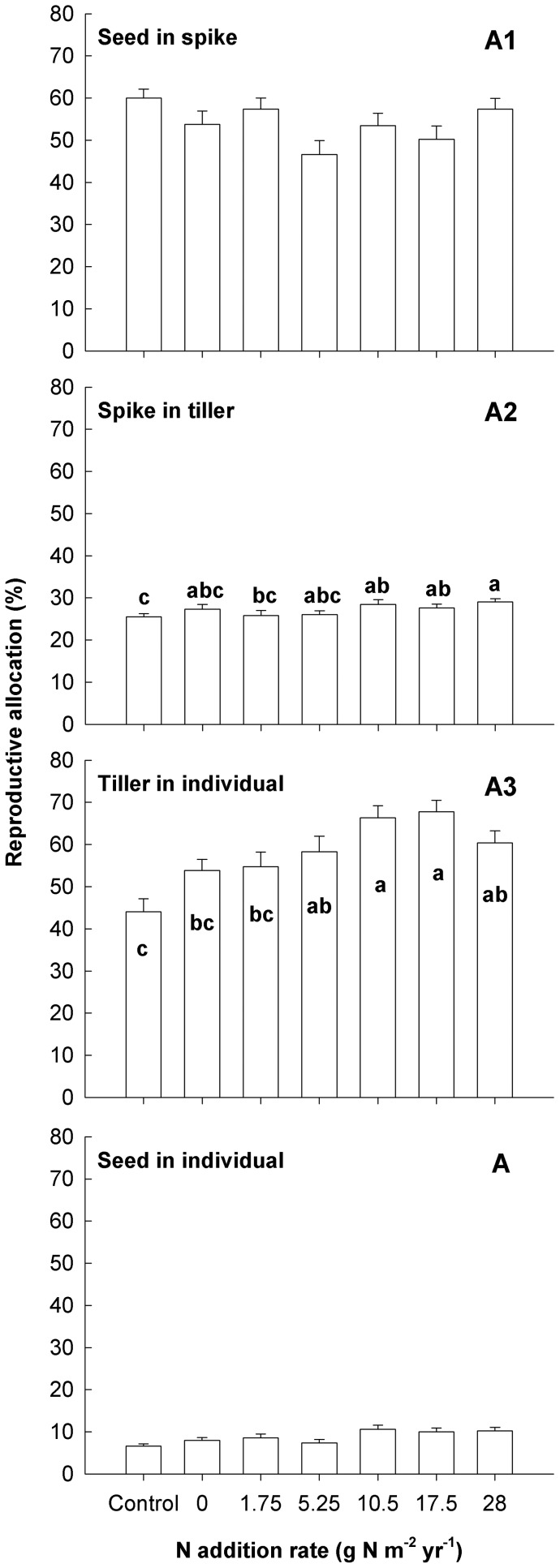
Effects of nutrient addition on biomass reproductive allocations (RAs) of *A. cristatum* at the spike (A1), tiller (A2) and individual (A3) levels and final RA (A) at the whole plant level. RA for each treatment is the average of 45 replicates (error bars indicate s.e.). Bars followed by the same letters are not significantly different according to LSMEANS at *P* = 0.05.

**Figure 6:**
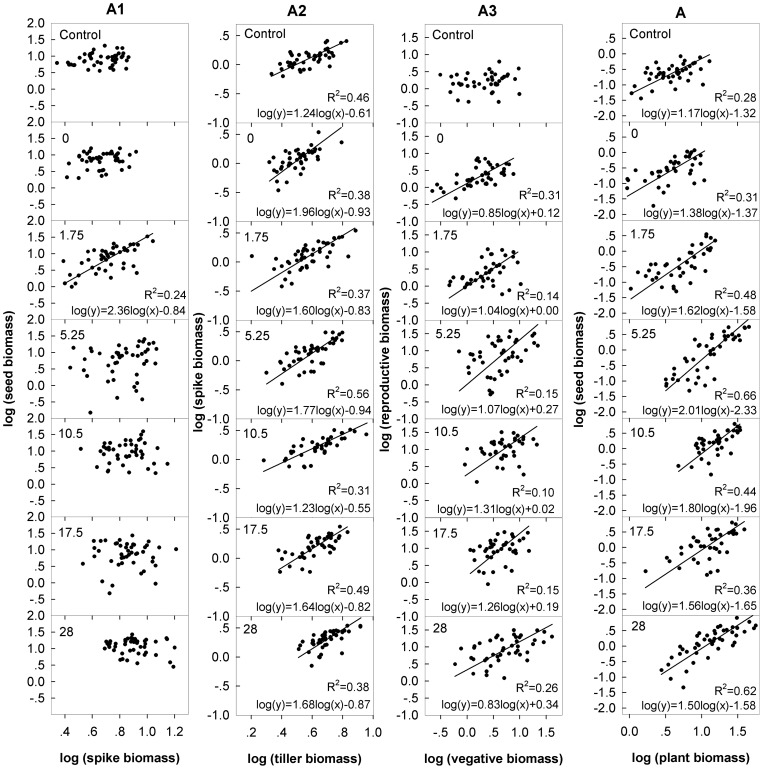
Allometric relationships of seeds vs. non-seed components at the spike level (A1), spike vs. non-spike components at the tiller level (A2), reproductive tillers vs. vegetative tillers at the individual level (A3) and seeds vs. non-seed components at the whole plant (A) level for control and N addition rates at 0, 1.75, 5.25, 10.5 and 28 g N m^−2^ yr^−1^. Subplots without regression indicates that there is no significant allometric relationship at *P* = 0.05.

**Table 2:** Effects of nutrient addition on the slopes and intercepts of reproductive allometric relationships at the spike (a1), tiller (a2) and individual (a3) levels and of the final R-V allometric relationship at the whole plant level (a).

log_10_Y vs. log_10_X	Nutrient addition rates (g m^−2^ yr^−1^)								
	Control	0	1.75		5.25		10.5	17.5	28
a1: seeds vs. non-seed components at the spike level (10^−2^ g)									
Slope	–	–	**2.36**			–	–	–	–
Intercept	–	–	−0.84			–	–	–	–
R^2^	–	–	0.24			–	–	–	–
Number of replicates	45	45	45			45	45	45	45
a2: spikes vs. non-spike components at the tiller level (10^−1^ g)									
Slope	1.24	**1.96**	**1.60**			**1.77**	1.23	**1.64**	**1.68**
Intercept	−0.61	−0.93	−0.83			−0.94	−0.55	−0.82	−0.87
R^2^	0.46	0.38	0.37			0.56	0.31	0.49	0.38
Number of replicates	45	45	45			45	45	45	45
a3: Reproductive tillers vs. vegetative tillers at the individual level (g)									
Slope	–	0.85		1.04		1.31	1.07	1.26	0.83
Intercept	–	0.12		0.00		0.02	0.27	0.19	0.34
R^2^	–	0.31		0.14		0.15	0.10	0.15	0.26
Number of replicates	45	45		45		45	45	45	45
a: Seeds vs. non-seed components at the whole plant level (g)									
Slope	1.17c	**1.38**bc		**1.62**abc		**2.01**a	**1.80**ab	**1.56**abc	**1.50**bc
Intercept	−1.32	−1.37		−1.58		−2.33	−1.96	−1.65	−1.58
R^2^	0.28	0.31		0.48		0.66	0.44	0.36	0.62
Number of replicates	45	45		45		45	45	45	45

Allometric slopes and intercepts were gained from the log–log linear relationship, log_10_ *Y*  = Slope×log_10_ *X*+Intercept, using regression SMA analyses. If the slopes of two allometric models were not significant, the effects of fertilization on the intercepts were tested. *R* ^2^ values represent how much variable percentage can be explained by the models. “–” indicates that the relationship is not significant at *P* = 0.05. Bold allometric slopes indicate that significant difference from 1 at *P* = 0.05. Figures with different letters are significantly different at *P* = 0.05.

### Target species

*Agropyron cristatum* is a widely distributed perennial bunchgrass in Eurasia grasslands. According to results of 31 years of monitoring work on species dynamics, the relative abundance and relative biomass of *A. cristatum* have increased significantly since 2005 (Figure S1). Moreover, in this study, it became a dominant species in the majority of plots after 11 years of nutrient addition. With increased levels of nutrient addition, its relative cover increased from about 30% in control plots to about 60% in the nutrient-added plots, and it accounted for about 45% of community-level aboveground net primary production (ANPP) in nutrient addition plots in 2010. This species displayed significant differences in ANPP among the treatments ranging from 68 to 208 g m^−2^ and with densities of reproductive individuals ranging from 7.45 to 12.31 plants m^−2^.

### Field sampling and measurement

At each plot, we examined the density, biomass and cover at community and species levels within a 1-m^2^ quadrat on 30 August 2010, which is near the end of the growing season and corresponds to peak biomass for most species. The number of reproductive individuals of *A. cristatum* was also recorded at that time. On 7 September 2010, when *A. cristatum* was fully mature, five reproductive individuals were randomly sampled in each plot and put into separate paper bags. In total, we collected 45 individuals for each treatment and 315 individuals for all treatments. In the laboratory, we separated the samples by the three levels of reproductive modules (Figure 1). An individual was separated into vegetative and reproductive tillers, a reproductive tiller was separated into spikes and non-spike components, and a spike was further divided into seeds and non-seed components. Five reproductive tillers from each individual were used to compare the RA variation at tiller and spike levels. All the separated components of the samples were dried at 65°C for 48 h and then weighed.

### Data analysis

The proportion of reproductive individuals (PRI) was calculated as follows: PRI (%)  =  the density of reproductive individuals in 1m^2^ / the density of total individuals in 1 m^2^. Plant size (aboveground biomass) frequency distributions were determined by dividing the range of individual plant sizes into 10 equal deciles. The effects of nutrient addition on PRI, population density and biomass were tested by one-way ANOVA with SPSS software (SPSS 11.0 for windows, USA). The differences between treatments were compared by Duncan's multiple range tests.

Based on our hierarchical sampling method, we assessed four types of RA within an individual. At the individual level, we calculated the fractions of reproductive tiller biomass in individual biomass. At the tiller level, we calculated the fractions of spike biomass in reproductive tiller biomass. At the spike level, we calculated the fractions of seed biomass in spike biomass. At the whole plant level, we determined the fractions of seed biomass in individual biomass. Accordingly, we examined the four types of reproductive allometric relationships.

Reproductive allometric relationships were analyzed by the common exponential model described as Y  =  βX^α^. In the present study, we log-transformed the equation as follows: log_10_ (Y)  =  log_10_ (β) + αlog_10_ (X); where X is the vegetative biomass (total biomass excluding biomass of reproductive structures) at different levels, Y corresponds to reproductive biomass, and α and β are the scaling slope and the allometric intercept, respectively [27], [28]. The Standardised Major Axis (SMA) regression measure was used to determine the slopes and intercepts by the software package (S) MATR (Standardised Major Axis Tests and Routines) [29], [30]. For each type of allometric relationship, the slopes were first tested to determine whether they were significantly different from 1. We then tested the difference in allometric slopes among treatments. If the slopes were significantly different, we did not test the difference between the intercepts; otherwise, the intercepts were further tested.

The effects of nutrient addition on sizes (individual biomass, reproductive tiller biomass and spike biomass) and RAs at three reproductive levels were analyzed by the PROC MIXED procedure of SAS with the REML (residual maximum likelihood) option with N treatment as the fixed effect and replicate and plant as random effects [31], [32]. Treatment means were separated with the LSMEANS statement [33].

## Results

### Population level response

There was no significant change in the density of *A. cristatum* when the N rate was lower than 5.25 g N m^−2^ y^r−1^, while a significant decrease was found when the N rate was higher than 10.5 g N m^−2^ yr^−1^. Population level biomass followed a unimodal pattern with a peak biomass achieved at a N rate of 5.25 g N m^−2^ yr^−1^. The proportion of reproductive individuals in *A. cristatum* displayed an increasing trend after nutrient addition and reached saturation when the N rate was higher than 5.25 g N m^−2^ yr^−1^ (Figure 2, Table S1).

### Size distribution and skewness

The patterns of plant size distribution differed significantly among seven nutrient addition treatments (Figure 3). The larger individuals increased significantly when the N rate was higher than 1.75 g N m^−2^ yr^−1^. Accordingly, the skewness decreased significantly with increasing rates of N addition.

### Biomass production at different hierarchical levels

Biomass production of *A. cristatum* at the three examined levels was significantly affected by nutrient addition (Table 1, Figure 4). There was a dramatic increase in biomass at the individual-level, while biomass increased gradually at the tiller- and spike-levels when the N rate was increased from 1.75 g N m^−2^ yr^−1^ to 5.25 g N m^−2^ yr^−1^. Moreover, biomass production at the individual level became saturated when the N rate was greater than 5.25 g N m^−2^ yr^−1^.

### Reproductive allocation at different hierarchical levels

With the increase in the rate of N addition, RAs both at the individual and tiller levels increased significantly (Table 1, Figure 5). However, RAs at the spike level were not significantly affected. RAs at the tiller level were the lowest among the allocations to reproduction at the three hierarchical levels. For the final RA (seeds vs. non-seed components) at the whole plant level, no significant difference among treatments was found.

### Reproductive allometric relationships at different hierarchical levels

At the spike level, no allometric relationship between seeds and non-seed components was found except for spikes harvested from the plots with nutrient added at a rate of 1.75 g N m^−2^ yr^−1^. At the tiller level, however, a fixed allometric relationship was found between spikes and non-spike components, as evidenced by a constant allometric slope. In contrast, at the individual level, a non-allometric relationship between reproductive and vegetative tillers in control plots shifted to fixed allometric relationships in nutrient addition plots. As an integrated outcome, the relationships between seeds and non-seed components at the whole plant level followed plastic allometric trajectories as indicated by varied slopes (Table 2, Figure 6).

## Discussion

### Reproductive allocation at different hierarchical levels

Our results demonstrate that RA patterns at different hierarchical levels within a perennial bunchgrass differ significantly in response to nutrient addition. A significant increase in RA following nutrient addition was found at the individual and tiller levels but not at the spike level, suggesting that the regulation of reproduction in *A. cristatum* is mainly carried out at individual and tiller levels. Because the duration of RA usually lasts about 100 d at the individual level, 70 d at the tiller level and 30 d at the spike level in Inner Mongolia grassland [34], the relatively longer durations of individual- and tiller-level RAs ensure the effectiveness of regulations in RA at both levels. Additionally, the stability in spike-level RA suggests that the regulation in RA at the spike level is weak for *A. cristatum*, and that the spike instead of the seed can be used to evaluate the reproductive allocation of this species.

Our study also revealed that RA at the tiller level was much lower than those at the individual and spike levels, suggesting that the tiller-level RA is likely to be a limiting factor in the process of reproduction in *A. cristatum*. This implies that a small change in tiller-level RA would translate into a large change in final RA at the whole plant level. In a previous study, we found that tiller-level regulation in RA is an important mechanism for *Stipa grandis*, a widely distributed perennial bunchgrass in Inner Mongolia grassland, in response to mowing disturbance [35]. Given that *A. cristatum* increased its tiller-level RA significantly following nutrient addition in the current study, tiller-level RA may be the most important factor in the regulation of RA for some grassland species in response to environmental changes in this area.

In an alpine grassland ecosystem, Niu *et al*. (2008) found that RAs in all plant species declined after 2 years of fertilization [36], which is consistent with some observations that plants under crowded conditions can usually increase their competitive ability for light by allocating more resources to leaves and stems at the expense of RA [37], [38]. In contrast, we found that the final RA in *A. cristatum* remained unchanged across all treatments. Two potential mechanisms may account for these contrasting responses in plant RA. First, nutrient addition usually enhances light competition between plant species [22], [39]. *A. cristatum* is taller than other species in the current community, thus giving this species an advantage in light competition. Second, the difference in plant density between the two communities may also be important. The plant density in the current community was about 200 plants m^−2^, while the density in alpine grassland community was 800–2000 plants m^−2^ [36]. Consistent with this explanation, Fortunel *et al*. (2009) found that among 18 herbaceous species, most RAs were hardly affected by nitrogen supply with a density of 100 plants m^−2^ [9]. These results indicate that the responses of plant RA to nutrient addition to a large extent depend on light conditions for the examined species.

### Size-dependent versus size-independent effects on RA at different hierarchical levels

No detailed study has evaluated size-dependent versus size-independent effects on plant RA at different hierarchical levels within a population. Our results revealed that the size-dependent effect and the size-independent effect may play different roles in plant RA at different hierarchical levels. At the spike level, the R-V relationships did not follow an allometric trajectory, suggesting that the size-independent effect plays a dominant role. In contrast, the R-V relationships followed a fixed allometric trajectory at the tiller level, implying that a size-dependent effect plays a primary role in regulating plant RA. At the individual level, we found a shift from a non-allometric relationship in control plots to a fixed allometric relationship following nutrient addition. These findings indicate that the dominant drivers in the variation of RA change from size-independent effects to size-dependent effects. Because the individual-level R-V relationship in all nutrient addition plots exhibited a fixed allometric relationship, a size-dependent effect is likely to be a major mechanism in regulation of plant RA at the individual level.

Size-dependent RA has been extensively examined in plant populations [1], [7]–[9]. Our results further demonstrate that the RA in *A. cristatum* is regulated by the size-dependent effect mainly at the individual and tiller levels. A size-dependent RA trajectory has been described as a bet hedging, low risk strategy [5], which ensures that the plant species converts certain plant growth to reproduction under changing environments to improve the species' fitness [7]. At the individual level, the allometric coefficiencies were not significantly different from 1 (except for the control treatment), which suggests that the increase in reproductive tiller biomass is linearly related with the increase in vegetative tiller biomass. Perennial grasses in Inner Mongolia grasslands often face drought stresses that at times lasts over a month. Under these conditions, a bet-hedging strategy in RA at the individual level is of great benefit to perennial species to balance the functions between survival and reproduction. For tiller level allometric RA, five out of seven coefficiencies were higher than 1, which suggests that more biomass was allocated to the spike than to vegetative organs within a reproductive tiller. This also indicates that once a bud of *A. cristatum* has developed into a reproductive tiller it will maximize its reproductive output.

In contrast to the RAs at the individual and tiller levels, the RAs at the spike level did not follow an allometric trajectory, suggesting that spikes of the same size differed substantially in seed production. This phenomenon may be due to selective abortion of some seeds within a spike, leading to significantly different seed production between spikes. Parent plants are selected to produce more homogeneous progenies within a spike; therefore, selective abortion by parents may reduce asymmetric competition between siblings [18]. In addition, if the resource is limited, the competition between siblings would be intensive, thus leading to differences in sizes of siblings [19]. Both mechanisms may result in larger variation in seed production of spikes of the same size. Moreover, the seed of *A. cristatum* is not only a reproductive organ it is also a storage organ; therefore, the spike-level seed mass may not be related with the mass of non-seed organs. Our results clearly indicate that size-independent effect was also a mechanism for the regulation of RA in *A. cristatum*. It has been suggested that size-independent changes in plant RA may be of benefit to species' evolution [5] and to buffering plant populations from environmental changes [4], [39].

More interestingly, the final R-V relationship (seeds vs. non-seed components) at the whole plant level followed a plastic allometric trajectory, suggesting that this relationship represents the integration of R-V relationships along hierarchical levels and the final RA at the whole plant level is determined by both size-dependent and size-independent effects.

Our study highlights the importance of hierarchical approaches in the analyses of plant reproductive allometric relationships. Our hierarchical analyses demonstrate that the regulation of RA in *A. cristatum* is mainly accomplished at the individual and tiller levels, and that a size-dependent effect plays an important role in regulating plant RA at both levels. These results indicate that these regulatory mechanisms operate in a controllable way. In short, our results demonstrate that a hierarchical analysis is essential for better understanding how a plant population regulates its RA, particularly when plants are exposed to changing environments.

## Conclusion

This study, to the best of our knowledge, is the first to examine plant RA and reproductive allometry along hierarchical reproductive levels within a plant population. Our results indicate that nutrient addition mainly affected the allocation patterns of *A. cristatum* at the individual and tiller levels. The distribution of plant size significantly changed, and a size-dependent effect is a dominant mechanism in the regulation of plant RA at both levels. Our results also demonstrate that the process associated with RA at the tiller-level may be one of the most important factors in the regulation of RA. These findings have important implications for our understanding of the mechanisms underlying plant responses in RA to future eutrophication resulting from human activities. In addition, we have developed a method of hierarchical analysis for revealing the underlying mechanisms responsible for variation in RA for perennial bunchgrass.

## Supporting Information

Figure S1 **Relative abundance (A) and relative biomass (B) of** ***A. cristatum*** **in Inner Mongolia grassland from 1980 to 2010.** Relative abundance  =  plant density of *A. cristatum*/plant density of the community. Relative biomass  =  plant biomass of *A. cristatum* /plant biomass of the community.
(TIF)

Table S1 **F values and** ***P*** **values of one-way ANOVA analysis of variance for the effects of nutrient addition on density, biomass and proportion of reproductive individual (PRI) of** ***A. cristatum*** **at the population level.**
(DOC)
