# Supplementary figures and images for: Hierarchical Reproductive Allocation and Allometry within a Perennial Bunchgrass after 11 Years of Nutrient Addition
Source: PLoS One. 2012 Sep 11;7(9):e42833. doi: 10.1371/journal.pone.0042833 (PMC3439474; doi:10.1371/journal.pone.0042833)

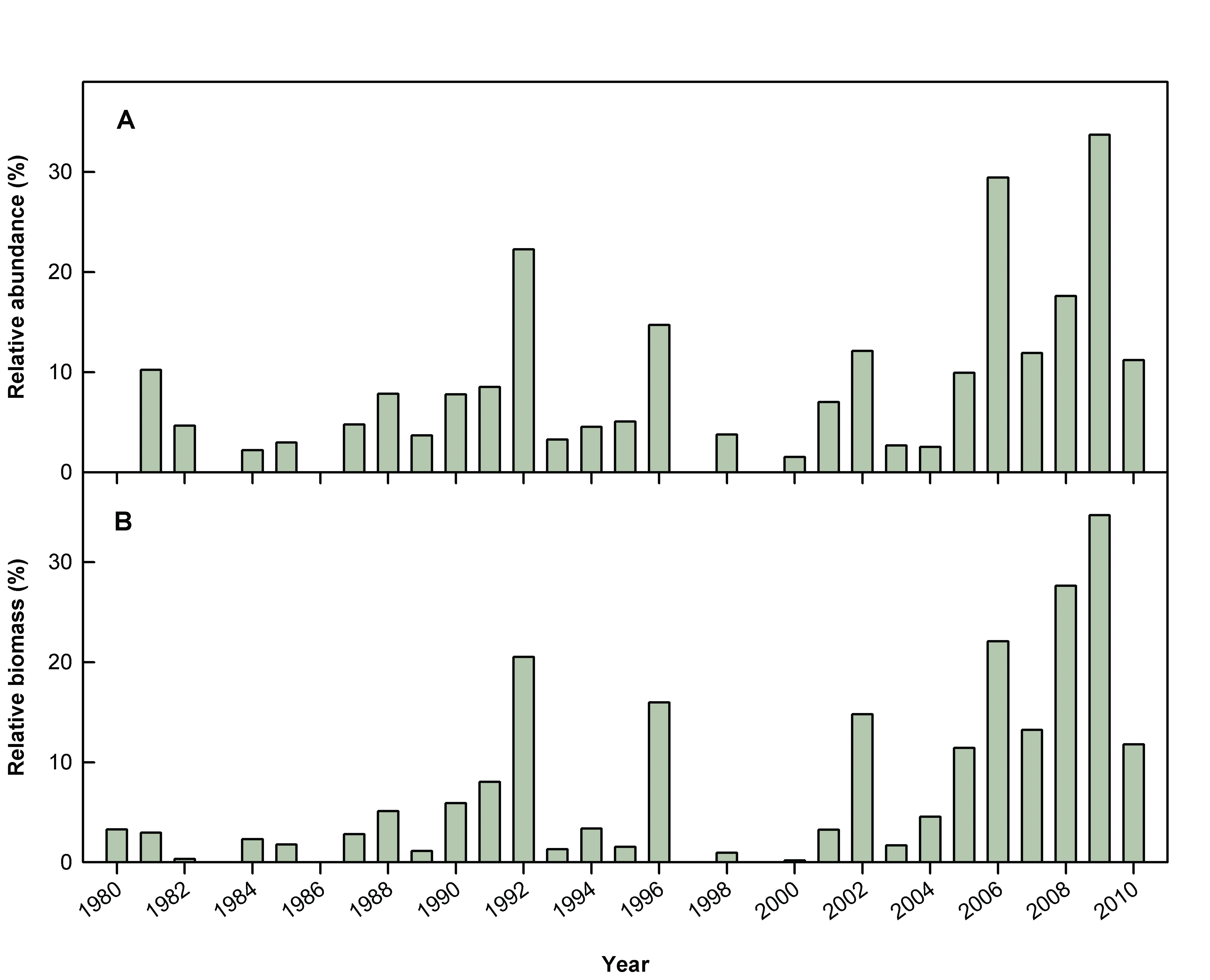

Supplement: Figure S1 — Relative abundance (A) and relative biomass (B) of A. cristatum in Inner Mongolia grassland from 1980 to 2010. Relative abundance = plant density of A. cristatum/plant density of the community. Relative biomass = plant biomass of A. cristatum /plant biomass of the community. (TIF) [file pone.0042833.s001.tif]
