# Supplementary material for: Hierarchical Reproductive Allocation and Allometry within a Perennial Bunchgrass after 11 Years of Nutrient Addition
Source: PLoS One. 2012 Sep 11;7(9):e42833. doi: 10.1371/journal.pone.0042833 (PMC3439474; doi:10.1371/journal.pone.0042833)
Supplement: Table S1 — F values and P values of one-way ANOVA analysis of variance for the effects of nutrient addition on density, biomass and proportion of reproductive individual (PRI) of A. cristatum at the population level. (DOC) [file pone.0042833.s002.doc]

Table S1. F values and *P* values of one-way ANOVA analysis of variance for the effects of nutrient addition on density, biomass and proportion of reproductive individual (PRI) of *A. cristatum* at the population level.

| Response variables | N | df | F value | *P* value |
| --- | --- | --- | --- | --- |
| Density (plants m^-2^) | 9 | 6 | 2.316 | 0.046 |
| Biomass (g m^-2^) | 9 | 6 | 5.086 | <0.001 |
| PRI (%) | 9 | 6 | 10.907 | <0.001 |
